# Managing Status Epilepticus in the Older Adult

**DOI:** 10.3390/jcm5050053

**Published:** 2016-05-11

**Authors:** Stephane Legriel, Gretchen M. Brophy

**Affiliations:** 1 Medico-Surgical Intensive Care Department, Centre Hospitalier de Versailles—Site André Mignot, 177 Rue de Versailles, 78150 Le Chesnay Cedex, France; 2 INSERM U970, Paris Cardiovascular Research Center, 75015 Paris, France; 3 Virginia Commonwealth University, Medical College of Virginia Campus, 410 N. 12th Street, Richmond, VA 23298-0533, USA; gbrophy@vcu.edu

**Keywords:** status epilepticus, anticonvulsant, elderly, older adult, adverse effects

## Abstract

The aim of this systematic review was to describe particularities in epidemiology, outcome, and management modalities in the older adult population with status epilepticus. There is a higher incidence of status epilepticus in the older adult population, and it commonly has a nonconvulsive presentation. Diagnosis in this population may be difficult and requires an unrestricted use of EEG. Short and long term associated-mortality are high, and age over 60 years is an independent factor associated with poor outcome. Stroke (acute or remote symptomatic), miscellaneous metabolic causes, dementia, infections hypoxemia, and brain injury are among the main causes of status epilepticus occurrence in this age category. The use of anticonvulsive agents can be problematic as well. Thus, it is important to take into account the specific aspects related to the pharmacokinetic and pharmacodynamic changes in older critically-ill adults. Beyond these precautions, the management may be identical to that of the younger adult, including prompt initiation of symptomatic and anticonvulsant therapies, and a broad and thorough etiological investigation. Such management strategies may improve the vital and functional prognosis of these patients, while maintaining a high overall quality of care.

## 1. Introduction

Status epilepticus (SE) is a major medical condition that is fatal in about 20% of cases [1]. The incidence per 100,000 population has been estimated at 9.9 episodes in Europe and 41 episodes in the US [2]. The aging population and comorbidities associated with this age class make management strategies for SE increasingly important. Unfortunately, these patients received relatively little attention. The aim of this systematic review is to bring a higher awareness to the important aspects of epidemiology, management modalities, and outcome in the older adult population with SE. 

## 2. Materials and Methods

Data reporting in this systematic review of SE in the older adult population is in accordance with the recommendations included in the PRISMA statement. Accordingly, the review question was formulated to respond to the following points of the PICO template: “In older adult patients who experience SE (P), are there any important aspects of epidemiology or management modalities (I), as compared with younger adult population (C), that may explain SE occurrence and outcome (O)?” Given the question being addressed, the retained eligible study designs were randomized and observational, controlled trials of older adult patients with SE.

### 2.1. Definitions

Status epilepticus was defined according to the the “Guidelines for the evaluation and management of status epilepticus”, from the Status Epilepticus Neurocritical Care Society Guideline Writing Committee, as an ongoing clinical and/or electroencephalographic seizure activity lasting at least 5 min, or repeating seizure activity without recovery (return to baseline) between attacks [3]. There is a high variability of definitions of the older adult population across the world; therefore, we used the definition of the World Health Organization definition based on age and distinguishing young-old (65 to 74 years old), middle-old (75–84 years old), and oldest-old (over 85 years old) [4].

### 2.2. Eligibility Criteria

This review focuses on studies describing and evaluating three types of outcome predictors: epidemiological characteristics, management modalities, and clinical outcomes. Since we were interested in describing particularities of SE occurrence in the older adult populstion and determine outcomes in this population, two types of comparators were used: (1) patients with SE under and ≥65 years of age, and survivors and non-survivors in the older adult (≥65 years of age) SE population.

All studies including older adult (≥65 years) patients who experienced SE were considered for inclusion. Patients with postanoxic SE were excluded from the review process. Prognostic studies in which the neurological outcome was described using either of the following scores, Glasgow Outcomes Scale (GOS) or the modified Rankin Scale (mRS), were included in the review. A favorable outcome was defined as a GOS score of 4 or 5, or a mRS score of 1 to 3. Outcome studies were included if the patients were assessed at hospital discharge, or greater than or equal to three months after discharge, when available.

### 2.3. Search Strategy

We searched MEDLINE via PUBMED using the following terms: “status epilepticus [MeSH Terms]” and “Aged [MeSH Terms]” or “elderly” or “older”. In order to maintain an updated search strategy, we activated an automatic PUBMED alert system from the first article selection to the last search round performed on 18 October 2015. To ensure that all potentially relevant articles were included, the reference lists of relevant review articles and articles selected for inclusion in this review were searched manually for other potential studies.

### 2.4. Study Selection and Data Extraction

Randomized and observational, controlled trials of adults 65 years of age or older with SE on indexed journals were included. There were no language or date restrictions for the published literature included in this review. Studies were selected and screened by titles and abstracts to identify studies reporting any of the selected interventions and outcomes of focus. Data extraction was performed using a dedicated form. The following data were extracted for each study: first author, publication year, study design, sample size, and primary and secondary outcomes including timing of evaluation.

## 3. Results and Discussion

**Figure 1:**
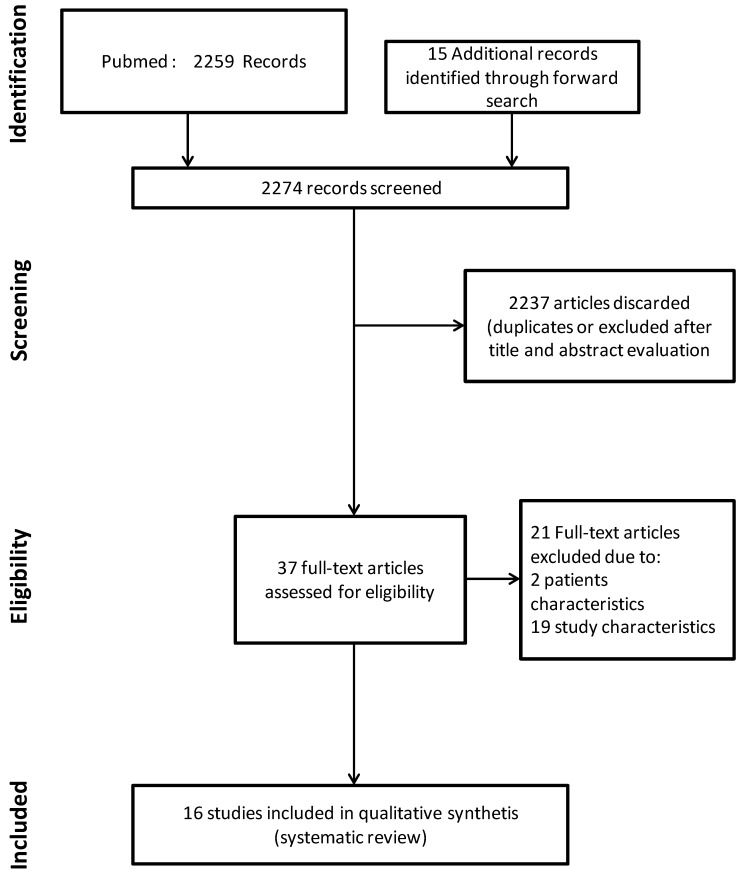
Flow chart of the study selection process.

Figure 1 is the flow chart of the study selection process. The literature search strategy identified 2259 records from PUBMED. Fifteen additional records were identified through forward search for a total of 2274 records screened. After title and abstract evaluation, 37 articles were considered for full-text analysis. Among them, 21 were excluded because they did not fulfil our inclusion criteria. The remaining 16 articles were considered eligible for this review. 

### 3.1. What Is the Epidemiology of Status Epilepticus in the Older Adults?

#### 3.1.1. Incidence

**Figure 2:**
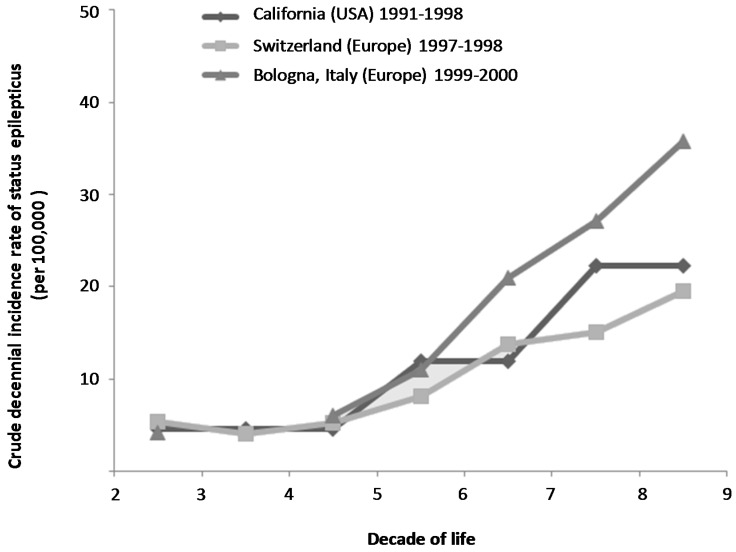
Age-specific crude decennial incidence in patients with status epilepticus in North America and Europe.

The WHO definition of older adults based on age seems to be particularly adapted to the context of epilepsy. A straightforward increase in incidence is effectively noted for every 5 years beyond 60 years old in this pathology [5,6,7]. The various epidemiological studies performed in populations of North American or European patients with SE report a similar trend, with a cut off after 60 years (Figure 2). Thus, mean annual incidence rate can be estimated to 15.5/100,000 in patients between 60–69 years old, 21.5/100,000 in patients between 70–79 years old, and 25.9/100,000 in patients over 80 years old [8,9,10].

#### 3.1.2. Classification

**Figure 3:**
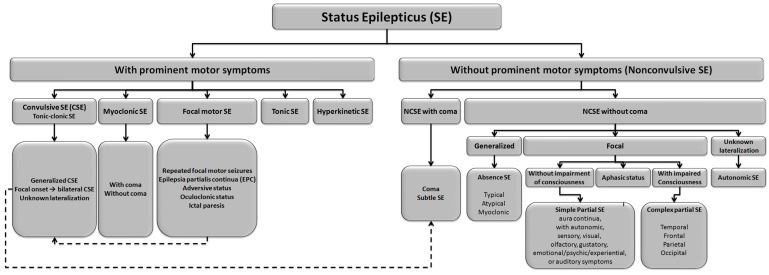
Classification of status epilepticus (adapted from the report of the ILAE Task Force on Classification of Status Epilepticus [12]).

The most widely accepted classification of SE is a pragmatic and operational scheme distinguishing between convulsive status epilepticus (CSE), which is usually easy to recognize on clinical grounds, and nonconvulsive status epilepticus (NCSE), in which the behavioral and/or cognitive changes persist as compared to baseline and where EEG confirmation is mandatory [11]. Subgroups are described within each of these two main categories. Figure 3 illustrates the various subgroups categories as described by the recent report of the ILAE Task Force on Classification of Status Epilepticus [12].

In the older adult population, there is an over-representation of complex partial NCSE [13,14]. In a study involving 63 patients over 70 years of age hospitalized in geriatric medicine for SE, Canouï-Poitrine *et al.* [13] identified 83% of complex partial NCSE, while the remaining 17% patients demonstrated CSE immediately or secondarily generalized SE. The cerebral distribution complex partial NCSE were as follow: fronto-temporal in 74%, temporal in 13%, and frontal and occipital 9% in 4% of cases, respectively [13].

#### 3.1.3. Mortality, Morbidity, and Determinants of Outcome after SE in the Older Adult

Mortality at hospital discharge after status epilepticus increases gradually with age and status epilepticus severity [15,16]. Whereas the mortality rate is about 13% in young adults, it reaches 38% in older adults of 60–79 years old, and was found up to 50% after 80 years [17]. Regarding severity of status epilepticus, mortality has been demonstrated as higher in patients with refractory status epilepticus [18] or super refractory status epilepticus aged over 75 years [19]. Independent predictors of mortality are also particularly marked by age since 65 years old has been identified as a fatal cut off value in several studies [17,18,20,21,22,23]. Others factors associated with hospital mortality are related to seizure duration, an underlying CNS structural lesion, *de novo* status epilepticus, intensity of consciousness disorders at scene and refractory status epilepticus [20,21,22,24,25]. Morbidity is also impacted in older adult survivors after status epilepticus. In a case control study of adults aged over 70 years hospitalized in a geriatric acute care unit, patients who experienced a status epilepticus episode significantly demonstrated functional impairment at hospital discharge than the others, in 85% and 69%, respectively [13].

Finally, long term outcome in patients who initially survived a first episode of status epilepticus is also clearly worse in older adults, demonstrating a 10-year mortality rate of 82% in a population of patients over 65 years *versus* 32% in young adults [26].

### 3.2. How Should Status Epilepticus Be Managed in the Older Adult Patient?

#### 3.2.1. Diagnosis of Status Epilepticus

The diagnostic strategy of status epilepticus is simple and does not differ in the older adult population. Most forms of CSE do not require EEG confirmation, except myoclonic seizures in particular cases (e.g., drug intoxication, post anoxic status epilepticus). The EEG is essential for the diagnosis of NCSE [27]. The diagnosis is based on the combination of a suggestive context, characteristic EEG patterns, and clinical response to treatment [27,28,29,30].

#### 3.2.2. Differential Diagnosis of Status Epilepticus in the Older Adult

In the older adult, the neurosensory manifestations of NCSE deserve special attention, as they may be mistaken for psychiatric disorders (e.g., mood disturbances, cortical blindness, mutism and impaired verbal fluency, echolalia, confabulation, behavioural disorders, dissociative psychosis, and psychosensory disorders). Thus, in this particular population, the first differential diagnosis that should be evoked in case of delirium, stupor, or even coma, is SE [27,28]. It is therefore important to perform an electroencephalogram systematically in this context since it identifies SE in 16% of cases [31].

Conversely, many forms of abnormal motor activity may be confused with convulsive SE (e.g., tetany, neuroleptic malignant syndrome, shivering, drug-induced myoclonus, decerebration posturing, hemiballism, athetosis, and limb shaking in patients with arterial stenosis). Other medical conditions can also mimic SE in the older adult such as syncope, low cerebral blood flow, stroke, migraine, drug intoxication, infections, metabolic disorders, sleep disorders, paroxystic memory disorders, or even dementia [32,33,34].

Pseudo-seizure is another interesting differential diagnosis. It is defined as paroxysmal motor or behavioral symptoms that simulate SE in the absence of detectable electrical seizure activity or identified brain lesions. Prolonged episodes of pseudo-seizures define pseudo-SE, which mimics SE. The incidence of pseudo-seizure in patients with known epilepsy is about 15% [35]. Of 85 patients with pseudo-seizure, 78% reported at least one episode of pseudo-SE and 27% ICU admission for pseudo-SE. Among the distinctive features of pseudo-SE that have been identified, eye opening and closing may be the best clinical feature for differentiating pseudo-SE from SE. Whereas eye opening is the rule during epileptic seizures (positive predictive value [PPV], 97%), the eyes are closed in most pseudo-epileptic seizures (PPV, 94.3%) [36]. Finally, older patients over 55 years can represent about 10% of all cases of pseudo-seizures. When compared with earlier pseudo-seizures onset, older patients no demonstrated significant differences in clinical semiology but were less likely associated with antecedent sexual abuse, more likely to have multiple comorbidities and to health-related traumatic experiences ” [37].

Errors in diagnosis can also be related to the recording and interpretation of the electroencephalogram. In addition to the artifacts inherent in the EEG recording technique, EEG patterns can be mistakenly ascribed to NCSE including periodic lateralized epileptiform discharges, bilateral periodic epileptiform discharges, generalized periodic epileptiform discharges, and triphasic waves, whose epileptic nature remains widely debated. These patterns should be interpreted with caution based on the clinical setting [30,38].

#### 3.2.3. Predictors of SE Occurrence in the Older Adult

**Figure 4:**
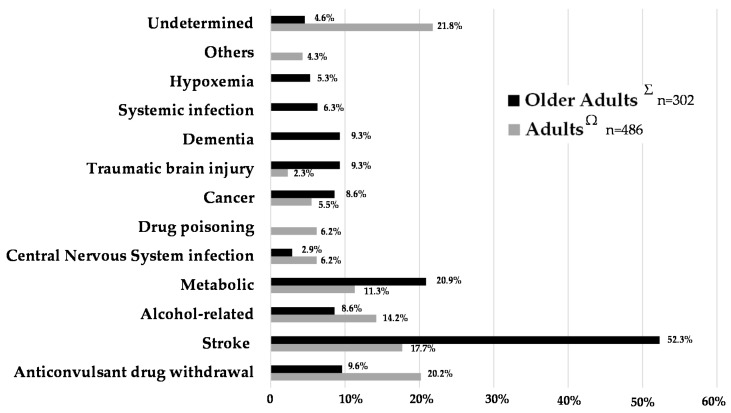
Comparison of causes of status epilepticus in 486 adults and 302 older adult patients. ^Ω^ Aminoff [40], Legriel [22] and Legriel [23]; ^Σ^ Sung [39], DeLorenzo [41], Canouï-Poitrine [13].

In doubtful cases, the combination of suggestive clinical manifestations and presence of factors frequently associated with status epilepticus in the older adults can reinforce diagnostic suspicion. A previous diagnosis of epilepsy and presence of a chronic neurological disease seem intuitively obvious. A recent study also reported a significant association of SE occurrence with the underlying presence of a dementia (other than neurovascular), an acute medical condition (cardiac, respiratory, or liver), or a dysnatremia [13]. Finally, combining several information sources, the main identified causes in the older adult are dominated by stroke (acute or remote symptomatic), miscellaneous metabolic causes, dementia, infection, hypoxemia, and brain injury (Figure 4) [13,17,39].

#### 3.2.4. Therapeutic Management of SE in the Older Adult

The paucity of studies dealing with management of status epilepticus in older adults with life threatening complications is an obstacle to the development of treatment strategies supported by a systematic review design. The only focus on this population was provided by Treiman and Walker who published a subgroup analysis from the Veterans study [42]. However, given the incidence of SE in the older adult, the relative contribution of patients aged over 65 years in studies dealing with SE is important, allowing us to consider the extrapolation of their results [43]. Thus, evidence is limited to support the following strategies herein proposed by the authors that are adapted according to guidelines management in the adult taking into account common particularities in the older adults.

Also, the severity of the presentation of SE in older adults requires urgent support based on the recommendations in adults regardless of age, while observing certain precautions and therapeutic choices guided by the context. The initiation of aggressive treatments must be balanced against the expected side effects of these treatments and natural history of the underlying disease treated according to its potential for neurotoxicity [14].

Etiological investigations should be carried out earlier in parallel to symptomatic and anticonvulsant treatment. Anticonvulsive treatment should be administered with progressive therapeutic escalations, taking into account the type of SE and response to prior treatments, with the final objective to definitively control seizure activity in up to 60 min from the onset of SE. Routine monitoring of anticonvulsant concentrations for agents with defined therapeutic targets is highly recommended to guide therapy and reduce the risk of toxicity.

In cases of failure of vital functions, hemodynamic stability should be ensured, particularly as many of the drugs used to treat SE can induce hypotension and/or heart failure. Catecholamine may be needed when using anesthetics in patients with refractory SE. The need for upper airway protection should be evaluated continuously while bearing in mind that the primary treatment goal is seizure resolution with recovery of consciousness. Therefore, an initial phase of coma without life-threatening manifestations is acceptable if not unduly prolonged. Considering endotracheal intubation should be particularly thought in the older adults by weighing the pros and cons. If it is performed, rapid-sequence induction may be preferred using etomidate rather than propofol or thiopental in order to avoid inducing cardiac failure. Succinylcholine can be used, provided there is no evidence of hyperkalemia. Hypoglycemia should be looked for routinely and corrected. If glucose is given, 100 mg of thiamine should be administered concomitantly, most notably when there is evidence of vitamin B1 deficiency. Patients should be routinely evaluated for hyperthermia and metabolic disturbances, which require prompt correction. Metabolic and/or respiratory acidosis should be controlled, and tests for acute renal failure with rhabdomyolysis should be performed. Aspiration pneumonia may complicate the initial consciousness disorders. Patients should be evaluated for injuries such as head injury and shoulder dislocation [44].

#### 3.2.5. Therapeutic Considerations in the Older Adults

In the acute phase of SE, it is important to consider therapeutic particularities related to age because there are changes in the pharmacokinetics and pharmacodynamics resulting in a significant inter-individual variability [45,46]. Thus, older adults are characterized by an alteration of gut or intestinal absorption, but also with an alteration of lipid and water distribution volumes, reducing distribution of medications based on these pharmacological properties. They also demonstrate a decrease in protein binding to albumin, increasing the free fraction of therapeutic agents which can then diffuse more easily beyond the blood brain barrier. Phenytoin and valproate are two anticonvulsants of concern due to approximately 90% protein binding [47]. Older adults also suffer from numerous comorbidities. Thus, respiratory and/or cardiac insufficiency may limit the use of some anticonvulsants. The combination of underlying neurological disorders can make these patients more susceptible to central depressant effects of certain therapeutic agents. Finally, renal or hepatic insufficiency can indicate or incite against particular caution in the use of certain agents (e.g., levetiracetam and renal dysfunction, sodium valproate and hepatic dysfunction) [45,46,48,49].

Older adults exhibit a higher potential for drug interactions given the number of concomitant medications [50]. Thus, one can encounter problems of enzyme induction of cytochrome P450 enzymes and iso IA2, 2B6, 2C9, 3A4/5 and uridine 5′diphospho glucuronosyl tranferases (UGT 1 and 2) (e.g., phenytoin and phenobarbital) that increase hepatic metabolism other treatments [46,51]. It may also pose the problem of inhibition of glucuronidation with cytochrome P450 and CYP2C9 (e.g., valproate) [46,51]. Interestingly, levetiracetam and lacosamide have the important advantage of not causing enzymatic induction or inhibition [48].

In the chronic phase, the relay of these treatments is difficult for some older adult patients [52]. There is a potential long-term use of some enzyme-inducing AEDs, which can impact other medication concentrations during the dose titration phase. It is therefore necessary to titrate anticonvulsant doses slowly and monitor serum concentrations (if available) for efficacy and toxicity. It is also important to simplify therapy as much as possible to improve adherence. Finally, use of newer anticonvulsants that have minimal adverse drug effects and drug-drug interactions should be considered [53].

#### 3.2.6. Treatment Strategies in Status Epilepticus

Anticonvulsant treatments appropriate for the electrical and clinical seizure pattern in the older adult patient should be initiated.

It is important to remember that a first single seizure with a duration of less than 5 min does not always require emergent treatment, but measures of supportive care and surveillance. Decision to maintain anticonvulsant medication should be more based on presence of risk factors for seizure recurrence rather than older age [54]. This point is particularly important in respect to older adult patients who may have more prolonged adverse effects from anticonvulsant medications. 

Once the diagnosis of SE has been made, the first line of treatment (emergent treatment) is to use benzodiazepines. These therapeutics can be administered by intramuscular, rectal, buccal, or intranasal routes when the intravenous route is not available. While intravenous lorazepam was previously considered the first-line treatment of reference, intramuscular midazolam demonstrated equivalence or even superiority in a recent study [55]. Extrapolation of this result to the population of older adult patients is, however, difficult since 80% of patients included were aged less than 60 years. Other agents may be diazepam or clonazepam whose use may be possible through the parenteral or intrarectal route [56,57,58]. It is important to note that midazolam has also been studied by the intranasal and buccal routes, which is potentially useful in older adult patients in who venous access is sometimes difficult.

One of the key studies in SE compared four anticonvulsants and referring treatments in tonic-clonic SE at generalized overt and subtle stages, with 44% of these patients being greater than 65 years of age [59]. Lorazepam and phenobarbital were both significantly better than phenytoin alone in this study. The results of the analysis by subgroups in the population of patients over 65 years showed an accentuation of efficacy differences described above, while it was not possible to achieve any statistical analysis in this subgroup population. It is also interesting to note that the time to first anticonvulsant treatment was longer in the older adult population, resulting in less efficacy of anticonvulsant treatments, even more pronounced when patients were found at scene in subtle SE (versus overt stage of SE) [42,43].

There are several choices for the second-line (urgent) treatment of SE. Based on the evidence, phenytoin/fosphenytoin or valproate would theoretically be the agents of choice. Levetiracetam and lacosamide are some interesting alternatives to consider, while phenobarbital is generally not a favorable option in older adult patients. Therefore, older adult SE treatment should be guided by the adverse drug effects of available anticonvulsant treatments. Phenytoin/fosphenytoin and lacosamide should be used with caution in patients with cardiovascular comorbidities, while phenobarbital has greater central and respiratory depression. Intravenous phenytoin and phenobarbital also contain a large amount of propylene glycol and may cause hemodynamic instability with rapid infusions. Valproate is contraindicated in cases of liver impairment, and finally lower doses of levetiracetam and lacosamide should be used based on reduced renal function in older adult patients. A recent review recommended levetiracetam dosing adjustment regimen according to creatinine clearance as follows: 500–1000 mg every 12 h in case of creatinine clearance between 50–80 mL/min/1.73 m^2^; 250–750 mg every 12 h in case of creatinine clearance between 30–50 mL/min/1.73 m^2^; 250–500 every 12 h in case of creatinine clearance <30 mL/min/1.73 m^2^ and 500–1000 every 24 h in case of end-stage renal disease. A 250–500 mg levetiracetam supplemental dose is recommended after each dialysis [60]. Lacosamide dosing adjustment regimen would not be necessary if creatinine clearance remains >30 mL/min/1.73 m^2^ [61]. In cases of severely impaired renal function, the maximum recommended dose is 300 mg with dosage adjustments according to creatinine clearance as follows: 150 mg every 24 h in case of creatinine clearance between 15–30 mL/min/1.73 m^2^; and 75 mg every 24 h in case of creatinine clearance <15 mL/min/1.73 m^2^. A supplemental dose of 25–150 mg (up to 50% of the current dose) of lacosamide is recommended after each dialysis [61]. In addition, dosage adjustments should be considered for lacosamide in patients with mild to moderate hepatic dysfunction. Further reductions should be considered in patients with renal or hepatic dysfunction taking concomitant strong CYP3A4 and/or CYP2C9 enzyme inhibitors. Lacosamide should not be used in patients with severe hepatic impairment [62].

Finally, third line therapies are those of refractory status epilepticus (RSE). They rely on the use of anesthetic agents, namely propofol, thiopental, pentobarbital, or midazolam. Whereas available data are insufficient to prefer one of these anesthetics over another, especially in the population of older adult patients, the particularly half-life associated with thiopental and pentobarbital should discourage use of these drugs as a first choice. Regardless of the drug used, a weight based loading dose should be considered and additional dose titration at 3–5 min intervals under EEG monitoring with the goal of obtaining a burst-suppression pattern with suppression for 5–10 s. Once this goal is reached, a continuous infusion is given to maintain the burst-suppression pattern for 12–24 h. Boluses should be given if the burst-suppression pattern is lost before the pre-specified time; after the boluses, the continuous-infusion dose should be increased gradually. The treatment-discontinuation modalities vary across agents, in relation to the differences in their half-life values. A 20% reduction every 3 h is appropriate with propofol and a 50% decrease every 3 h with midazolam, whereas thiopental and possibly pentobarbital can be stopped with no prior dosage reduction. In patients that are difficult to control, slower withdrawal of RSE treatment should be considered. A loading dose of one or two long-acting antiepileptic agents should be given routinely in combination with the anesthetic agent and continued after anesthesia withdrawal [3].

#### 3.2.7. Etiological Investigations of Status Epilepticus in the Older Adult

In addition to these symptomatic and specific measures, etiological investigations should be promptly performed. Main causes of SE may differ in adult *versus* older adult populations. A rigorous initial clinical examination should be conducted and associated with the realization of diagnostic tests for diagnostic purposes. Hypoglycemia (or hyperglycemia) should be systematically investigated and corrected as well as hyperthermia and possible metabolic disorders (e.g., hypocalcemia, hyponatremia, high uremia, hypomagnesemia, hypoxemia, carbon monoxide, hypercapnia). A blood alcohol assay can be performed. Similarly, the search for subtherapeutic anticonvulsants should be systematically evaluated in the epileptic population. The search for other metabolic disorders (porphyria, thyroid dysfunction) or the search for toxic substances (cocaine, amphetamines, tricyclic/serotonergic antidepressants) will be based on the context [63]. We always raise the possibility of iatrogenic cause (overdose of beta-lactams, quinolones, isoniazid, theophylline, *etc.*). Among toxic causes, we should systematically look at elements associated with posterior leukoencephalopathy. In this same hypothesis, we will look for a hypertensive encephalopathy. Brain imaging is ideally performed on admission to not be disturbed by the initiation of a continuous EEG recording, and in order to enable faster management of mass lesions that need neurosurgical intervention. A brain scan without and with contrast should be routinely performed in the initial management of patients that do not regain consciousness. An MRI may also be considered if all the etiologic diagnosis remains negative. 

A lumbar puncture will also systematically be carried out in feverish context, if meningeal stiffness is observed or in immunocompromised patients, and in those whose etiologic remains negative. Given the suspicion of meningitis, encephalitis, or meningoencephalitis, systemic and CSF cultures should be obtained and antimicrobials initiated early and oriented toward suspected microorganisms. If neoplastic meningitis is suspected, lumbar puncture may be repeated up to three times to improve the diagnostic yield [44].

## 4. Conclusions

The management of SE in older adults requires attention because of increased incidence, some diagnosis difficulties, increased frailty, and a particularly poor outcome. The use of anticonvulsant drugs may be problematic in older adults. It is important to take into account the specificities related to the pharmacokinetic and pharmacodynamics changes: altered lipid and water distribution volumes resulting in lower distribution therapeutic agents involved, altered protein binding causing an increase in circulating serum levels of therapeutic agents normally bound to albumin, multiple comorbidities making it difficult to use certain treatments, and finally drug interactions related to the anticonvulsants with hepatic enzyme inducing or inhibiting properties. Beyond these precautions, the management may be identical to that of the younger adult, associating only prompt initiation of symptomatic and anticonvulsant treatments, and a broad and thorough etiological investigation. Such management strategies could improve the vital and functional prognosis of these older adult patients with SE. 

## Abbreviations

The following abbreviations are used in this manuscript:

Status Epilepticus (SE)
Glasgow Outcomes Scale (GOS)
Electroencephalogram (EEG)
Modified Rankin Scale (mRS)
Convulsive Status Epilepticus (CSE)
NonConvulsive Status Epilepticus (NCSE)
Positive Predictive Value (PPV)
